# A Perplexing Presentation of Entrapment of the Brachial Artery

**DOI:** 10.1155/2015/236193

**Published:** 2015-06-22

**Authors:** Deniz Cevirme, Eray Aksoy, Taylan Adademir, Hasan Sunar

**Affiliations:** ^1^Kartal Kosuyolu Education and Research Hospital, Department of Cardiovascular Surgery, 34862 Istanbul, Turkey; ^2^American Hospital, Department of Cardiovascular Surgery, 34365 Istanbul, Turkey

## Abstract

A 45-year-old male being otherwise healthy presented acute onset of right upper extremity ischemia. On physical examination, axillary artery could be palpated whereas the brachial artery could not be palpated below the level of the antecubital fossa, including radial and ulnar artery pulses. Pulses were also inaudible with pocket-ultrasound below the level of the brachial artery bifurcation. The patient was initially diagnosed to have acute thromboembolic occlusion and given 5000 IU intravenous heparin. The patient was taken to the operating room. We noticed that the ischemic symptoms disappeared within a couple of minutes just before we began the operation. However, ischemic symptoms reappeared six hours later and computed tomography angiography showed lack of enhancement below the elbow crease. We were taking the patient to the operating room for the second time when the symptoms recovered in a few minutes, again. The operation was not canceled anymore. In the operation, the brachial artery was found anomalously perforating and it was entrapped by the bicipital aponeurosis. The artery was relieved by resecting the aponeurosis and there was no need for any other intervention. The patient had no more recurrence of symptoms postoperatively.

## 1. Introduction

There have been several anatomical variations of the upper extremity vasculature in anatomy literature where ramification patterns were the most common. The brachial artery piercing the bicipital aponeurosis has been a rare finding in cadaveric studies and this type of variation was accompanied by the presence of a brachioulnar artery or, more infrequently, of a brachioulnoradial artery [1]. It is clear that variations in the upper extremity nerves and vessels may be troublesome during surgical practice.

The brachial artery entrapment is rare, may be associated with muscle or fascial hypertrophy, and may occur in individuals doing physically demanding jobs [2, 3]. We present here a patient who presented acute onset of right upper extremity ischemic symptoms which eventually was diagnosed as entrapment of the brachial artery in its course where it pierced the bicipital aponeurosis.

## 2. Case Report

**Figure 1:**
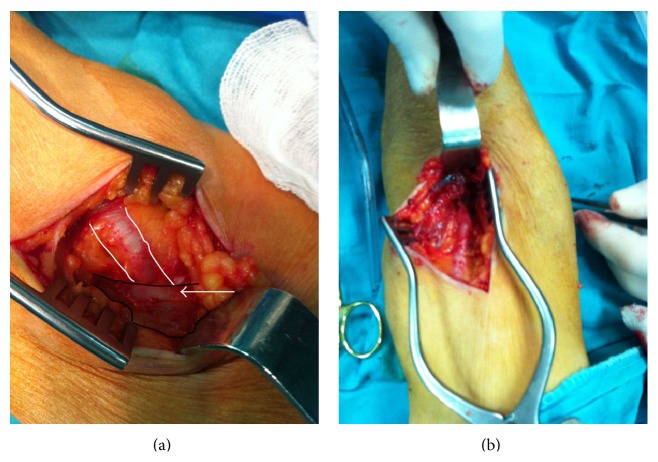
(a) The brachial artery (white borders) seen piercing through and entrapped by the bicipital aponeurosis (white arrow shows the aponeurosis—dark borders). (b) The brachial artery liberalized by resecting the aponeurosis.

A 45-year-old male presented newly developed pain, coldness, and numbness of the right upper extremity. The pain gradually increased and he said that he could not move his right hand and fingers for about 1 hour. He never had such complaint before. He denied having been exposed to any trauma and he had no previous history of cardiac, vascular, rheumatological, and neurological diseases and intervertebral herniation. The right arm was cold and right hand and fingers were cyanotic on inspection. The axillary artery could be palpated whereas the pulse of the brachial artery was found to have disappeared below the level of antecubital fossa. Radial and ulnar pulses could not be palpated. Cardiac and lung auscultation was normal and lower extremity and left upper extremity arteries could be palpated. Electrocardiogram showed a normal sinus rhythm without any other abnormal finding. Initial workup included total blood count and blood urea, creatinine, liver enzymes, and routine ELISA panel all of which were found within normal limits. Arterial flow dynamics were evaluated using a pocket-ultrasound device. A normal triphasic waveform was audible above the level of the brachial artery whereas the waveform was biphasic below that level and inaudible at the level of ulnar and radial arteries. Based on these findings, the initial diagnosis was acute thromboembolic occlusion of the right brachial artery. An initial 5000 U of intravenous heparin was given. The patient was immediately taken to the operating room for thrombectomy. Interestingly, ischemic symptoms suddenly improved during the preparation for the operation. The cyanosis degraded and radial and ulnar pulses became palpable. The patient said that his arm and hand have been almost completely relieved and warmed. The operation was canceled. Computed tomography angiography was considered for differential diagnosis of aortic dissection. However, the patient denied giving consent for use of intravenous contrast medium when he was informed about the risk of contrast nephropathy and hypersensitivity. The patient was initiated on intravenous heparin infusion (1000 IU per hour). Symptoms suddenly reappeared six hours later, but this time the cyanosis of the right arm was more severe. The patient gave consent for the radiographic evaluation and contrasted tomography was performed. Contrast enhancement was found normal on proximal segments of the right upper extremity arterial bed; however, it was found to have totally disappeared just above the level of ulnar-radial artery bifurcation. While the patient was being immediately taken to the operating room, symptoms suddenly disappeared again; cyanosis degraded and the hand became warmed while his distal pulses became palpable, again. Local anesthesia was made with subepidermally applied lidocaine 10% and a longitudinal incision was made over the antecubital fossa. The brachial artery was explored deep in the fossa and was seen anomalously perforating the bicipital aponeurosis. It was found constricted by both bicipital tendon and bicipital aponeurosis (Figure 1(a)). The aponeurosis and surrounding tissues were removed using sharp dissections and the artery was liberalized (Figure 1(b)). Because the radial and ulnar pulses were palpable and the ischemic signs and symptoms totally disappeared, further intervention was not performed. Postoperative course was uneventful. The patient had no more recurrence of symptoms and was discharged on the day after the operation.

## 3. Discussion

Entrapment of the brachial artery is an infrequent entity and was first described in a 29-year-old male in 1977 [2]. Clinical presentation may manifest as acute progression of ischemic symptoms or the progression of symptoms may be interrupted by intermittent relief periods. The surrounding peripheral nerves may also be compressed. Bassett III et al. [3] reported that 5 patients with forearm ischemia and pain were successfully treated; the pathology was hypertrophy of the bicipital aponeurosis. It was reported that variations may exist in insertion site of the pronator teres muscle and such anomalies may cause entrapment in individuals doing physically demanding jobs. Median nerve may also be involved in such conditions [4]. Acute arterial ischemia is an emergent condition which should immediately be fixed even though the definitive cause of the condition could be established. Also, variations of the brachial artery may be associated with risk of thrombosis [5, 6]. However, the brachial artery rarely has an anatomical variation whereas bicipital aponeurosis had an anomalous structure. The patient did not suffer from disability of right upper extremity movements after the operation. The authors recommend that entrapment of the brachial artery with surrounding structures should be kept in mind in patients presenting acute intermittent upper extremity ischemic symptoms.
